# 5-fluorouracil-induced coronary vasospasm: A cardiovascular magnetic resonance imaging case report

**DOI:** 10.21542/gcsp.2023.16

**Published:** 2023-08-01

**Authors:** Kelly Garbis, Moezedin Javad Rafiee, Judy Luu

**Affiliations:** Department of Medicine, Division of Cardiology, McGill University Health Center, McGill University, Montreal, Quebec, Canada

## Abstract

We report the case of a 45-year old male with metastatic colon cancer who presented with chest pain and transient diffuse ST-segment elevation on electrocardiogram after his third cycle of FOLFOX (folinic acid, fluorouracil, oxaliplatin). Initial transthoracic echocardiogram showed reduced left ventricular ejection fraction of 35% with mildly elevated troponins. Further investigations with cardiovascular magnetic resonance imaging demonstrated recovery of left ventricular function with evidence suggestive of coronary vasospasm. This case report will review the utility of cardiovascular magnetic imaging in the evaluation of underlying etiologies for myocardial injury in patients with low likelihood of obstructive coronary artery disease.

## Case

Certain agents frequently used in patients with active neoplasms, such as anthracyclines or HER-2 inhibitors, are commonly recognized for their cardiotoxicity. Fluoropyrimidines have also frequently been associated with cardiotoxic effects. These antimetabolites, including capecitabine, floxuridine, and 5-fluorouracil (5-FU), are commonly used chemotherapeutic agents, particularly for gastrointestinal malignancies. Numerous studies have described variability in the incidence of 5-FU associated cardiotoxicity (0–35%)^1^. The clinical presentation may vary, from myocardial ischemia, arrhythmias, cardiogenic shock, to sudden cardiac arrest^1,2^.

We describe a case of 5-FU induced coronary vasospasm, confirmed on CMR, in a patient with stage IV colon cancer presenting as myocardial ischemia and new-onset LV systolic dysfunction.

A 45-year-old male with no previous cardiac history, and known for metastatic colon cancer, presented with new-onset chest pain two days after his third cycle of FOLFOX. Upon initial investigation, the electrocardiogram (ECG) showed atrial fibrillation with rapid ventricular response. Diffuse ST-segment elevation was also found on the anterolateral leads of the ECG. Acute ST segment elevation myocardial infarction (STEMI) was suspected, and the cardiology service was promptly contacted. After complete evaluation, the working diagnosis was of possible pericarditis and the patient was not sent for cardiac catheterization. Initial troponin levels were elevated at 146 ng/L (0–53 ng/L), with a peak reaching 258 ng/L. After stabilization, the patient was transferred to our local hospital for further management.

**Figure 1.**
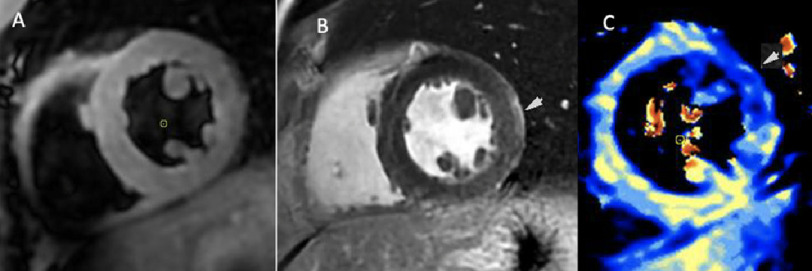
CMR views. A) T2-weight short-tau inversion recovery (STIR) left ventricular mid short axis (SAX) image shows increased signal intensity of the myocardium due to edema. B) Late gadolinium enhancement (LGE) SAX view shows linear subepicardial enhancement in the mid anterolateral segment. C) T2-weighted map shows globally increased T2-values (blue), predominantly in the lateral wall, indicative of myocardial edema.

At our tertiary center, transthoracic echocardiography (TTE) revealed global left ventricular (LV) hypokinesia with an estimated left ventricular ejection fraction (LVEF) of 35%. The myocardial appearance and longitudinal strain patterns were suggestive of an infiltrative process. In view of these results, cardiac magnetic resonance (CMR) was obtained eight days after initial presentation.

CMR imaging revealed an LVEF of 61% with minimal hypokinesia of the distal cavity. On late gadolinium enhancement (LGE), there was suspicion of a linear, subepicardial region of enhancement along the mid-inferolateral, anterolateral, and inferior segments. Myocardial T2 values were diffusely increased, predominantly in the mid- to distal lateral wall, which was suggestive of edema (Figure 1). In the context of recovered LV function with LGE and myocardial edema, the conclusion was in favor of coronary vasospasm with a reversible myocardial injury.

Following the CMR results showing improved LV systolic function, the patient was maintained on angiotensin receptor blockers, beta-blockers, and oral anticoagulants for atrial fibrillation. The patient was safely discharged with outpatient follow-up with cardio-oncology.

## Discussion

Fluoropyrimidine-induced cardiotoxicity has been described, but many aspects remain poorly understood. Presentations may vary from arrhythmias to cardiovascular collapse and death^2,3^. The mechanisms described are multifactorial and include vasospasm in the context of endothelial dysfunction and direct cardiomyocyte injury^3^. Patients often present with chest pain and ECG changes suggestive of ischemia, which typically occur within a few days of administration of the chemotherapeutic agent^3^. Continuous infusions in comparison with bolus infusions may be associated with greater cardiotoxicity^3^. A recent study suggested that patients tend to be younger and do not always have traditional cardiovascular risk factors^4^. However, there are no clear guidelines for its management. Discontinuation of the offending agent is initially recommended^2^. If continued fluoropyrimidine use is necessary, vasodilator therapy with nitrates and calcium channel blockers should be administered to decrease the risk of recurrence^2^.

Our patient presented with several features of cardiotoxicity following FOLFOX chemotherapy. He presented with new-onset chest pain in close temporal relationship to the infusion of fluorouracil. He also presented with a new arrhythmia (atrial fibrillation), as well as ECG changes and troponin level rise suggestive of acute myocardial injury; two reported clinical presentations of fluorouracil toxicity. Although our patient did not undergo coronary angiography, acute plaque rupture was considered less likely based on the absence of traditional risk factors for coronary artery disease. The rapid recovery of LV systolic function, presence of myocardial edema, and subepicardial LGE without evidence of ischemic scarring on CMR were all suggestive of a non-ischemic etiology^5^. Given the diffuse ST-segment elevations upon initial presentation and the absence of ischemic scarring on CMR, we are confident that the patient did not experience STEMI. Based on pertinent negatives on multimodality imaging, other etiologies of myocardial injury in patients with suspected non-obstructive coronary arteries, such as thromboembolism, myocarditis, and Takotsubo cardiomyopathy (TCM), were considered less likely. Although TCM with rapid recovery of LV systolic function may not be completely excluded, the localized area of edema may be more favorable for coronary vasospasm. Consequently, coronary vasospasm after 5-FU infusion was the strongest diagnosis.

## What have we learned?

Fluorouracil infusion may be associated with chest pain, LV dysfunction, and ST-segment changes, suggesting an acute myocardial infarction. These symptoms are usually present in a close temporal relationship with chemotherapy infusion. Cardiotoxicity secondary to fluorouracil infusion may include coronary vasospasm and arrhythmias such as atrial fibrillation, as seen in our case. Thus, coronary vasospasm should be considered in the differential diagnosis of chest pain and suspected acute coronary syndrome in patients presenting with 5-FU infusion. CMR can be helpful in determining the underlying etiology of myocardial injury (elevated troponin levels) without suspected obstructive coronary artery disease.
